# Land use and land cover changes drive ecosystem services value in the Chinese county of Qianyang

**DOI:** 10.1038/s41598-025-29320-8

**Published:** 2025-12-12

**Authors:** Wang Shuangao, Pedro Cabral, Yuefeng Xu, Felipe S. Campos

**Affiliations:** 1 Beijing Academy of Science and Technology, No.27, Beike Building Xisanhuan North Road, Haidian District, Beijing, 100089 China; 2 NOVA Information Management School (NOVA IMS), Universidade Nova de Lisboa, Campus de Campolide, 1070-312 Lisboa, Portugal; 3 School of Remote Sensing and Geomatics Engineering, Nanjing University of Information Science and Technology, Nanjing, 210044 China; 4 School of Economics, Central University of Finance and Economics, Shahe campus, Changping District, Beijing, 102206 China; 5 Universitat Autònoma de Barcelona, 08193 Cerdanyola del Vallès, Spain; 6 Centre de Recerca Ecològica I Aplicacions Forestals (CREAF), 08193 Cerdanyola del Vallès, Spain

**Keywords:** Land use and land cover changes, Ecosystem services value, Landscape patterns, Environmental monitoring, PLUS model, Spatial autocorrelation, Environmental sciences, Environmental impact

## Abstract

Understanding how land use changes affect ecosystem services is essential for guiding sustainable development and environmental policy. In rapidly urbanizing regions, quantifying these impacts helps inform land management decisions. This study aims to analyze the impact of land use and land cover (LULC) changes on Ecosystem Services Value (ESV) in Qianyang County, China. We utilized land use data from 2007 to 2022 with a spatial resolution of 30 m. The spatial distribution and clustering of ESV were examined using spatial statistical methods. The Patch-generating Land Use Simulation (PLUS) model, which simulates LULC changes under three scenarios for 2050, was employed to calculate ESV and identify driving forces. Results show that urbanization and topography were the main driving factors for LULC changes in Qianyang County. Our findings provide valuable insights into sustainable land management and ecosystem conservation in the region.

**Supplementary Information:**

The online version contains supplementary material available at 10.1038/s41598-025-29320-8.

## Introduction

Anthropogenic pressure constitutes the primary driver affecting landscape patterns and ecosystem services (ES) provision^1^. Understanding this pressure, encompassing economic development, urbanization, industrialization, tourism growth, and overpopulation, is essential for formulating mitigation strategies to enhance environmental restoration^2^. As proposed by de Groot^3^, ES are vital for humanity^4,5^, as categorized by the Millennium Ecosystem Assessment^6^, are the benefits derived from ecosystem functioning, including supply, regulation, support, and cultural services for numerous environmental and socioeconomic activities. Hence, biodiversity conservation and development needs should be integrated into a united framework^7,8^. Furthermore, human activities can affect landscape patterns and the provision of other essential commodities and services^9^. However, landscape change and ES are interdependent^10^. Changes in landscape patterns, for instance, invariably result in substantial or minor modifications to ecosystem components, structure, ecological processes, and biodiversity, while also impacting the ES supply^11^. On the other hand, the degradation and loss of ES can have detrimental effects on the structure and aesthetics of landscapes, endangering regional and even global ecological integrity^12^. Consequently, monitoring the long-term changes of landscape patterns and the effects of ES can help us adapt to the changing trend of the regional ecological environment^13,14^. Thus, by evaluating observed trends for enhanced conservation objectives, we can rationally allocate and utilize land, improve local environmental quality, and promote the harmonious and sustainable development of humans and nature^15^.

China has had significant Gross Domestic Product (GDP) average growth (9.8%) over the previous 40 years (from 1980 to 2020), making it one of the world’s fastest-growing developing nations^16^. This economic growth, however, has come at the expense of natural ecosystems, causing degradation, habitat fragmentation, and resource depletion, which in turn undermine ES provision^17,18^. The well-being and health of the population were impacted by the rise in harmful environmental externalities caused by human-induced landscapes. To tackle this problem, the Chinese government developed initiatives, such as the Ecological Civilization Construction (2015–present) aligned with global frameworks like The Economics of Ecosystems and Biodiversity (TEEB)^19^. It also made the decision to adopt an ambitious plan of environmental sustainability and development in response to pressures to take environmental issues, such as soil erosion, biodiversity loss, and water resource degradation, into account^20,21^. These plans call for political changes in key sectors, such as agriculture, energy, and industry. The assessment of Ecosystem Service Values (ESV) has attracted plenty of attention^22^. The value of various ES has thus been the subject to an increasing number of research^23,24^.

There have been many studies on the value of ecological services in the past from the perspective of carbon emissions and alternative cost methods^25,26^. However, many of these studies focused on static ESV assessments, lacking dynamic simulations of future changes^27,28^. Additionally, while there have been various quantitative assessments and predictions of ESV^29,30^, county-level analyses like this one offer finer-resolution insights into local ecological dynamics. Over the past 30 years, ES research has evolved from static valuation to dynamic modeling and policy integration (e.g. Mapping and Assessment of Ecosystem Services—MAES^31^). This study contributes by linking LULC changes to ESV at a finer scale. The innovations in this study are multifaceted. Unlike most prior studies about ESV, which are carried out for large regions^30,32,33^, our study focuses on a small region, investigating a county-level unit, which allows for a more detailed and nuanced understanding of ESV. Secondly, this study analyses the continuous dynamic changes in ESV over time, providing a temporal perspective that is often missing in static assessments. Finally, this study delves into the reasons and mechanisms behind the changes in ESV, offering insights into the processes that influence ESV. This approach enhances ESV assessments by contributing to a more comprehensive understanding of ecosystem services and their dynamics.

The LULC changes significantly influence ESV, which are critical for sustainable land management and policy decision-making. The aim of this paper is to evaluate the LULC changes in Qianyang County, Shaanxi Province, China, focusing on ES supply and economic values, landscape dynamics and resource allocation in the region. This study provides a spatial distribution of ESV and their relationship with the ecological values’ coefficients of various land use types. The Coefficient of Sensitivity (CS)^29^ is used for cross-validation of ESV coefficients, ensuring the method’s reliability to determine the dependence of ESV over time on the coefficient value^34^. The ESV of each land cover class was classified and ranked, and spatial autocorrelation tools, such as Moran index and cold/hotspot analysis were applied to explore the spatial evolution processes at a regional scale over 15 years (2007–2022). Qianyang Country was selected because it has a dominant agricultural land in an impoverished mountainous region with a fragile natural environment, where the urban sprawl has been exacerbating due to the existing scarcity of natural resources. We explore the inter-annual LULC changes and their respective implications on ES provision. We quantify LULC-ESV dynamics using scenario simulation and driving factor analysis. This study differs from our previous work^35^, which focused on regional ESV assessment via landscape pattern changes and image fusion. For this paper, we employ the PLUS model to simulate LULC under three scenarios for 2050, enabling deeper analysis of ESV changes and driving forces.

## Materials and methods

### Study area

**Fig. 1:**
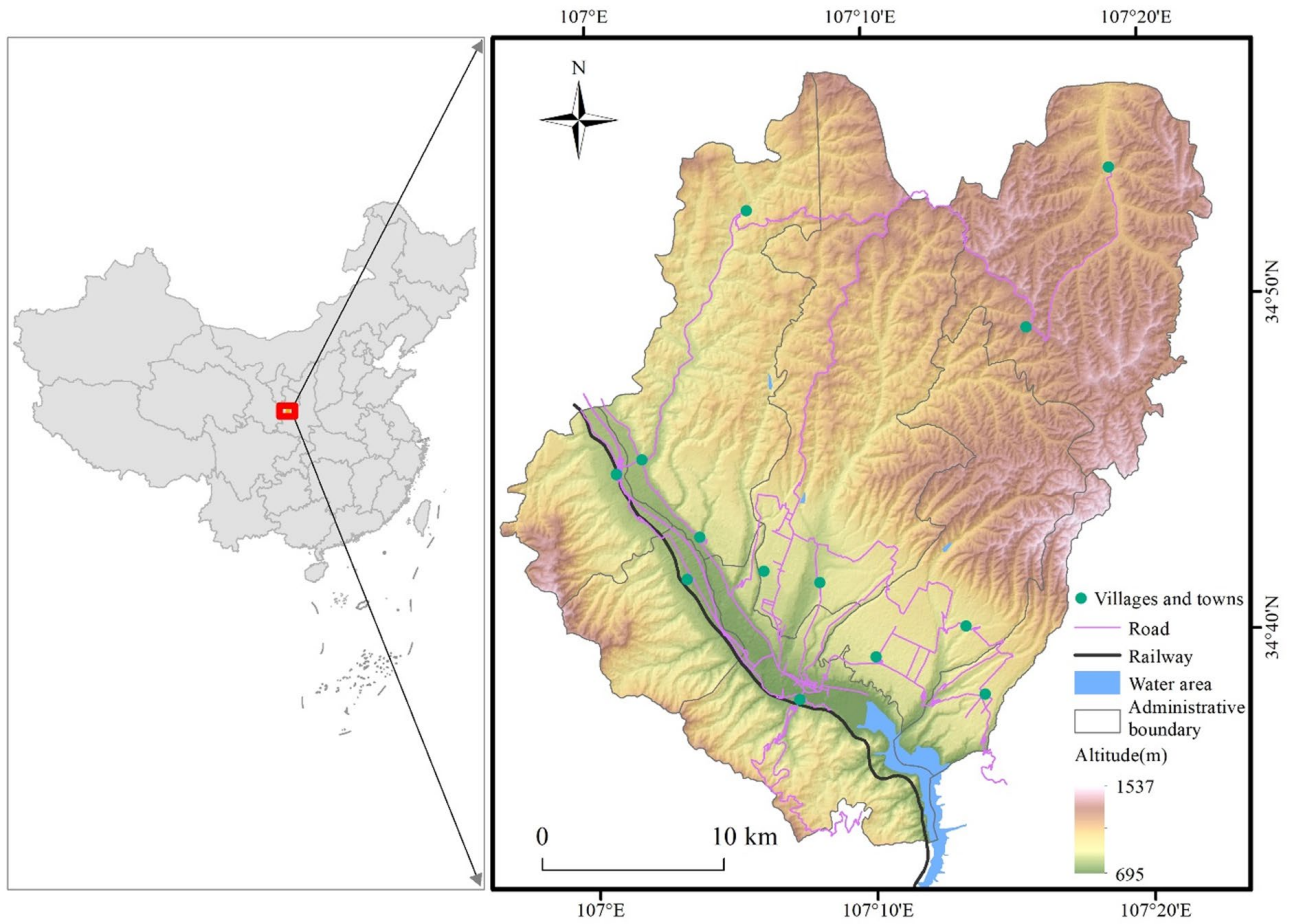
Study area location: Qianyang County, Shanxi Province, China. The administrative boundaries were obtained from the National Geographic Information Public Service Platform (https://map.tianditu.gov.cn). The maps were generated using ArcGIS Pro software version 3.2 (ESRI: https://www.esri.com/en-us/arcgis/products/arcgis-pro/overview).

Qianyang County is located in the hilly and gully region of the Weibei dry plateau, west of Shaanxi province (Fig. 1). The landform covers a total area of 996.46 km^2^ and includes seven hills, two tablelands, and one sub-river. The altitude ranges from 710 to 1545.5 m. The region has a sub-humid continental monsoon climate, with an annual average temperature of 10.9 °C and a 197-day frost-free season. The average annual precipitation is 677.1 mm. Among the many rivers in the area, the Wei River is the largest. Approximately 110,000 smallholder farmers cultivate most of the croplands, which span about 20 km^2^ ^36^.

### Data

This study focuses on the period from August 2007 to August 2022, with data acquisition conducted using the Geospatial Data Cloud (GSCloud, http://www.gscloud.cn/). Remote sensing images from the Landsat series, including Enhanced Thematic Mapper (ETM) and Operational Land Imager (OLI), were screened and downloaded. These sensors were selected for their multi-spectral observation capabilities, covering visible to near-infrared bands over the study area, providing high-resolution, long time-series data support for subsequent land use/land cover (LULC) monitoring and meeting the needs for dynamic evolution analysis of landscape patterns. Satellite imagery was interpreted using ENVI 5.3, with pre-processing including radiometric calibration (gain/offset parameters applied via the Radiometric Calibration tool) and atmospheric correction (FLAASH model).

As a fundamental step in remote sensing data preprocessing, radiometric calibration aims to eliminate systematic errors of the sensor. The software invokes radiometric calibration parameter files (containing key coefficients such as gain and offset, determined by satellite launch parties based on strict radiometric calibration field experiments) to convert the original digital number (DN) values of the images into radiance values, establishing a physical relationship between pixel values and real surface radiation energy, which provides accurate input for subsequent atmospheric correction.

The FLAASH (Fast Line-of-Sight Atmospheric Analysis of Spectral Hypercubes) atmospheric correction module integrated in ENVI 5.3 was used to further process the radiometrically calibrated images. During this operation, the appropriate atmospheric model (e.g., mid-latitude summer/winter standard atmospheric model) was selected in the software based on the geographical latitude of the study area (e.g., extracting the central latitude using administrative division vector data) and the imaging month of the image (corresponding to the season, such as the summer model for August images). Combined with the underlying surface characteristics of the study area, an urban aerosol model was selected to accurately simulate the influence of aerosol scattering if artificial surfaces such as construction areas existed, while a rural model was chosen for natural surfaces. Meanwhile, parameters such as the average altitude of the study area (obtained by statistics from Digital Elevation Model (DEM) data) and the sensor elevation angle (automatically read from image metadata) were input. The software corrected the radiation distortion caused by atmospheric scattering and absorption pixel by pixel based on radiative transfer theory and output the real surface reflectance image to maximize the restoration of ground object spectral characteristics.

Geometric registration is carried out to eliminate geometric deformation caused by satellite sensor attitude deviation, earth curvature and other factors, and ensure spatial consistency of images from different time phases and sensors. Firstly, from the images of the research area from 2007 to 2022, images with accurate geographic references and clear land features (such as including obvious road networks and river contours) were selected as benchmark images. Subsequently, using the ENVI 5.3 control point selection tool, Ground Control Points (GCPs) with the same name were collected on the reference image and the image to be registered through a combination of manual selection and automatic matching. Priority was given to selecting stable feature points such as road intersections, river turning points, and corner points of landmark buildings. The control points were required to cover the entire image uniformly, with a quantity set according to the image size and deformation degree, generally not less than 15–20, and the registration error was controlled within 0.5 pixels. After completing the collection of control points, a quadratic polynomial transformation model was used to fit the coordinate relationship of the control points and calculate the geometric transformation parameters. We used bilinear interpolation for resampling, assigning reasonable grayscale values to the deformed pixels, converting the image to be registered to the reference image coordinate system. This process enabled a spatial alignment of multi-source images, laying a spatial foundation for subsequent land use classification.

### Methods

**Fig. 2:**
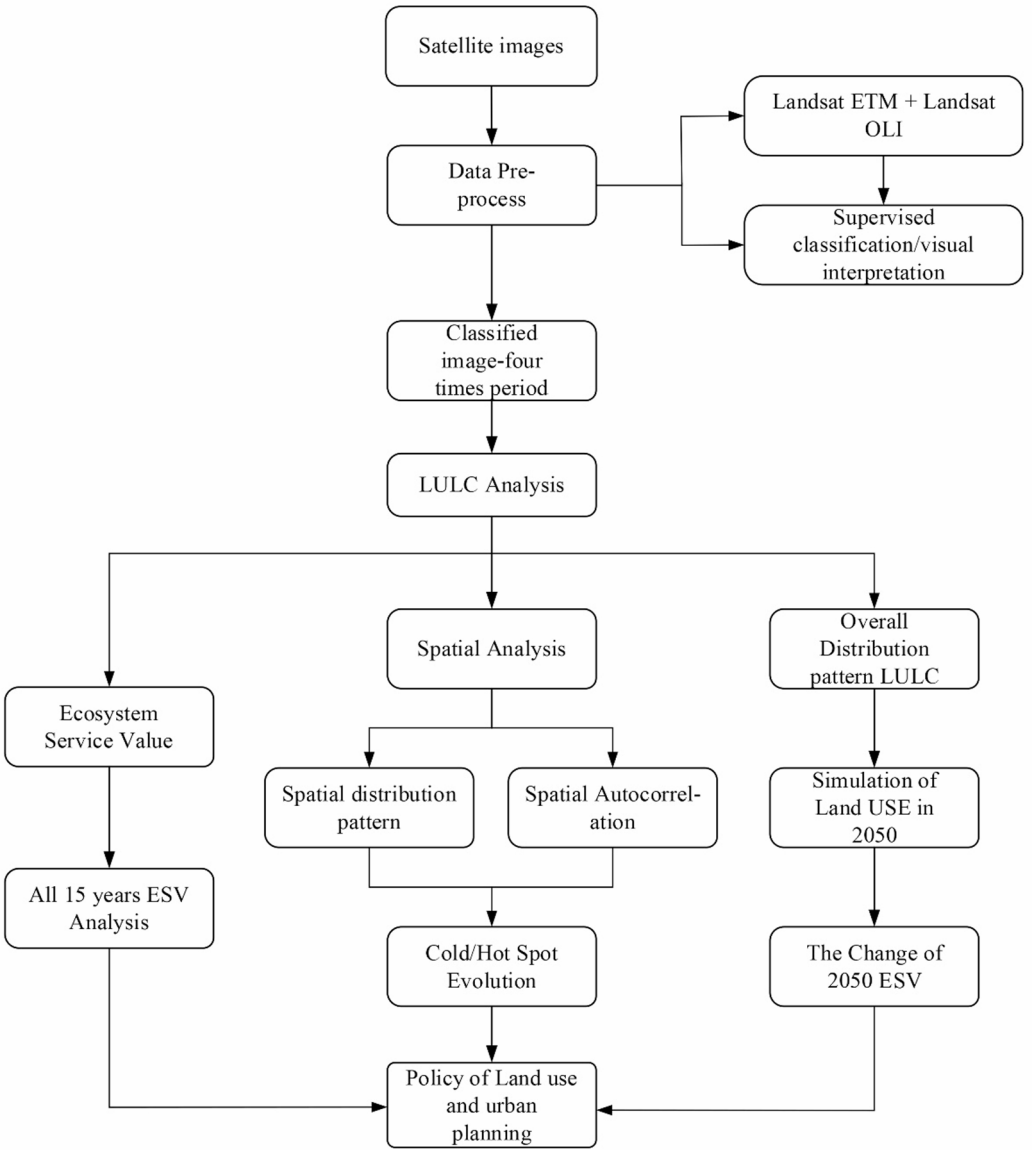
Methodological framework.

The methodological process used in this study is shown in Fig. 2. After preprocessing the land use data, we evaluated the ESV dynamics through historical trend analysis, spatial clustering, and scenario simulation by: (1) calculating and analyzing ESV over 15 years; (2) examining the ESV distribution through spatial clustering to identify hot and cold spots; and (3) using the PLUS model to simulate LULC under three scenarios for 2050 and identify the driving forces. We also assessed the accuracy of the results. To better understand the changes in ESV and LULC, we used cold/hot spots to display the evolution of Qianyang County. Hotspots refer to areas with statistically significant high ESV aggregation (e.g., forests and water bodies), while coldspots represent areas with significant low ESV clustering (e.g., construction lands).

#### LULC classification and accuracy assessment

Based on the preprocessed remote sensing images, a LULC classification system was constructed using a combination of visual interpretation and supervised classification. In the visual interpretation link, combined with high-resolution images for auxiliary interpretation (if available), interpretation marks were established for typical ground objects (such as the regular field texture of farmland and the continuous vegetation spectrum of forest land) according to the spectral, texture, and shape characteristics of ground objects. In supervised classification, training samples were selected, and algorithms such as the maximum likelihood method and support vector machine (configured as needed in ENVI 5.3) were used to classify image pixels, dividing the study area into six LULC types (cropland, forest, grassland, water body, building area, and unused land), following the GB/T 21010-2017 GB/T21010-2017, a standard aligned with regional land management practices, prioritizing compatibility with local policy datasets. After accuracy verification (such as confusion matrix test, calculating overall accuracy and Kappa coefficient), a classification thematic map was generated as the basic data layer for land use change analysis.

The supervised classification process was enhanced by integrating machine learning algorithms. Support Vector Machine (SVM), a robust supervised classifier, classifies pixels by mapping spectral data to a high-dimensional feature space via a radial basis function (RBF) kernel, with optimization of the penalty coefficient C to distinguish complex land covers (e.g., urban buildings and bare land). RF, as an ensemble learning method, constructs multiple decision trees (typically 50–200 trees) to improve robustness against spectral noise (e.g., cloud shadows), with parameters including the number of trees and feature variables per node (set as the square root of the spectral bands). Training samples were collected using ROI tools based on high-resolution imagery and field surveys, with no less than 100 pixels per class and K-fold cross-validation (K = 5/10) to split training (70%) and testing (30%) datasets.

The preliminary classification results may have issues such as misclassification, missed classification, and small spots, which require post-processing. This process involved using clustering analysis, majority/minority filtering, and other methods to smooth the classification results and remove small patches. Logical judgment and rule sets to correct unreasonable classification results, such as forcing non water area pixels located in rivers to be classified as water area pixels were also used. In addition, obvious misclassified areas were manually edited and corrected through visual interpretation.

We divided the spatial distribution of different land use types based on ESV. Spatial analysis techniques were adopted based on spatial autocorrelation to detect clustering effects. These images were pre-processed with ENVI 5.3 for radiometric and atmospheric correction, stitching, and cropping. Radiometric correction was performed using the Radiometric Calibration tool, while atmospheric correction was carried out with the FLAASH Atmospheric Correction model in ENVI 5.3, where the radiometrically corrected images received atmospheric correction^37^. Points of interest were selected by combining field data fixes and Google images. Eighty sample points were selected in each of the six categories (i.e., 1: Cropland, 2: Forest, 3: Grassland, 4: Water body, 5: Building area, and 6: Unused land. We used the SVM algorithm for classification, as it is considered one of the most robust classifiers^38^. After the interpretation was completed, the visual correction method was employed to rectify the misclassified land categories. The confusion matrix test was performed as one of the validation metrics.

The accuracy of LULC land classification needs to be determined by establishing an independent ground truth validation dataset, comparing the classification results with the truth to form a confusion matrix, and then calculating core indicators such as overall accuracy, producer accuracy, user accuracy, and Kappa coefficient. At the same time, spatial pattern accuracy can be considered by combining methods such as spatial autocorrelation analysis and patch scale accuracy evaluation. Its accuracy is affected by factors such as remote sensing image quality, sample representativeness, classification algorithm, and land classification definition. In actual evaluation, appropriate indicators need to be selected based on the characteristics of the land classification in the research area and application requirements. Generally, an overall accuracy of ≥ 85% and Kappa ≥ 0.8 are considered high-quality classification, and the accuracy of key land classifications needs to reach over 90% to ensure the reliability of classification results.

#### Land use dynamic degree model

This study adopts the land use dynamic degree model to analyse the speed of land use change in the study area^39^ (Eq. 1).

1 $$K = \frac{{U_{b} - U_{a} }}{{U_{a} }} \times \frac{1}{T} \times 100\%$$

where *K* represents the annual rate of change of a certain land use type in the study period, *U*_*a*_ and *U*_*b*_ represent the area of a certain land use type at the initial stage and the final stage of the study, respectively, and *T* represents the study period.

#### ESV assessment

Based on the ESV equivalent table per unit area of China’s terrestrial ecosystems^40^, ES were divided into four primary categories by MAES (1: Supply services; 2: Regulation services; 3: Supporting services; 4: Cultural services), and 11 secondary categories (1: Food production; 2: Raw material production; 3: Water supply; 4: Gas regulation; 5: Climate regulation; 6: Waste disposal; 7: Hydrological regulation; 8: Soil conservation; 9: Maintain nutrient; 10: Biodiversity; 11: Aesthetic landscape). The economic valuation of ES for urban areas was not estimated because its CS is extremely small, so the ESV of construction land in the classification of this study is 0. Hence, we adopted the calculation method of estimating the change of ESV in the study area as proposed by Costanza et al.^41^ and Xie et al.^42^, adjusting for regional biomes and 2007 Chinese Yuan (CNY) values. The ESV formula is as follows (Eq. 2):

2 $$ESV = \sum\limits_{k - 1}^{n} {A_{k} } \times V_{k}$$

where *ESV* represents the total value of ES, *A*_*k*_ represents the area of the *k-*land use type, and *V*_*k*_ represents the ESV coefficient of the *k-* land use type, that is, the *ESV* per unit area of each ecosystem.

#### ESV cold/hot regions

The Getis-Ord Gi* index^43,44^ identifies spatial clustering patterns of ESV changes. Hotspots represent areas with high ESV clustering (natural landscapes with forest, water bodies), while coldspots indicate areas with low ESV clustering (urban/construction areas). A higher Z-score of the Gi* index indicates dense high-ESV aggregation (hotspots), where surrounding values are significantly elevated—similar to population density hotspots in urban studies. Lower scores signify tight low-ESV clustering (coldspots). A Z-score near zero indicates non-significant clustering with random value distribution. Non-significant areas show no statistical ESV clustering (via Getis-Ord Gi*), consisting of mixed croplands/grasslands/scattered forests, where ESV is moderate and spatially heterogeneous, lacking the concentrated high/low values that define hotspots/coldspots. Ecologically, conservation efforts should prioritize maintaining/expanding hotspots and curbing coldspot (e.g., urban) expansion to sustain high ESV region. The calculation formula of *G*_I_^*^ index is follows (Eq. 3):

3 $$G_{i}^{*} = \frac{{\sum\limits_{j = 1}^{n} {W_{i,j} } x_{j} - \overline{x}\sum\limits_{i = 1}^{n} {W_{i,j} } }}{{\sqrt[s]{{\left[ {n\sum\limits_{j = 1}^{n} {W_{i,j}^{2} } - \left( {\sum\limits_{j = 1}^{n} {W_{i,j} } } \right)^{2} } \right]/(n - 1)}}}}$$

4 $$\overline{X} = \frac{1}{n}\sum\limits_{i = 1}^{n} {x_{i} } \quad S = \sqrt{\frac{1}{n}} i = \sum\limits_{i = 1}^{n} {x_{i}^{2} } - (x)^{ - 2}$$

where *x* is the mean of ESV; *x*_*j*_ is the ESV exponential change (DESV) of spatial unit *j*; *W*_*x,j*_ is the binary space weight matrix; *n* is the number of spatial units.

#### PLUS model

The PLUS model incorporates a rule mining framework utilizing the Land Expansion Analysis Strategy (LEAS) and a cellular automaton model employing multi-class random seeds (CARS)^45^. The LEAS module identifies areas of change for each land use type using two periods of land use data. It then calculates the impact of various driving factors on the expansion of each land use type by employing sampling methods and ratios, in conjunction with the random forest algorithm integrated into the PLUS model. The probability of development for each land use type is determined, providing a more comprehensive understanding of the factors and mechanisms that drive changes in land use. The CARS module relies on the initial land use data and the likelihood of development for each land use category. To improve the simulation of changes in land use types at the patch level and the spatial distribution pattern of future land use, it is necessary to establish appropriate parameters such as neighborhood weights, land transfer cost matrices, and the number of patches needed for each land use type^46^.Random forest parameters were configured^45^, with a sampling rate of 0.01, selecting about 1% of pixels for training to ensure accuracy and efficiency. The number of regression trees was set to 20, and the mTry value was 10, aligning the number of features with the driving factors. To enhance computational speed, the number of parallel threads increased to 18. These settings allowed us to determine each factor’s contribution (see Fig. 6), providing a crucial basis for further simulations. Three scenarios were designed: (1) Natural Development (business-as-usual)^47^, (2) Ecological Protection (restricted urban expansion)^48^, and (3) Urban Priority (accelerated construction)^49^, reflecting policy trade-offs.

## Results

### Land use/Land cover classification accuracy assessment

The Kappa indices of all land use types (Table 1) reached a value above 0.9100. The user and overall accuracy were above 83% and 90%, respectively, which is considered a highly satisfying accuracy metric value^50,51^. The Kappa statistic is over 90% (Table 2), indicating that the accuracy assessment results are acceptable. As shown in Table 3, the accuracy for all six land categories and the overall accuracy exceeds 80%, providing a convincing outcome for the next steps.

**Table 1:** Accuracy assessment of the classified maps for 2005, 2010, 2015 and 2020.

Year	Users accuracy	Overall accuracy	Kappa
2007	87.64	94.74%	0.91
2012	91.56	95.99%	0.93
2017	86.64	97.04%	0.95
2022	83.65	96.99%	0.95

**Table 2:** Accuracy of assimilated datasets with respect to Landsat reference image (RMSE is the Root Mean Square Error).

Band	Correlation coefficient	Reflectance RMSE
Green	0.92	1.64
Red	0.93	1.38
Blue	0.95	1.85

**Table 3:** Accuracy assessment values for the LULC images by 2007, 2012, 2017, and 2022.

Classes	2007		2012		2017		2022	
	PA	UA	PA	UA	PA	UA	PA	UA
Building area	82.83	87.10	98.63	93.10	87.86	91.77	89.26	91.62
Forest	95.88	99.65	97.31	99.73	98.19	99.87	98.05	97.46
Cropland	80.09	80.22	86.43	80.12	81.82	87.08	81.43	84.65
Unused land	80.38	87.65	88.85	83.35	86.93	89.69	86.19	84.43
Water body	93.85	96.93	96.95	98.62	95.03	99.38	94.28	95.41
Grassland	87.28	90.54	89.97	93.04	87.85	94.53	91.07	87.72
Overall accuracy	87.64		91.56		86.64		83.65	
Kappa	0.9138		0.9339		0.9511		0.9502	

PA, producer accuracy; UA, user accuracy.

### Land use/Land cover changes

**Fig. 3:**
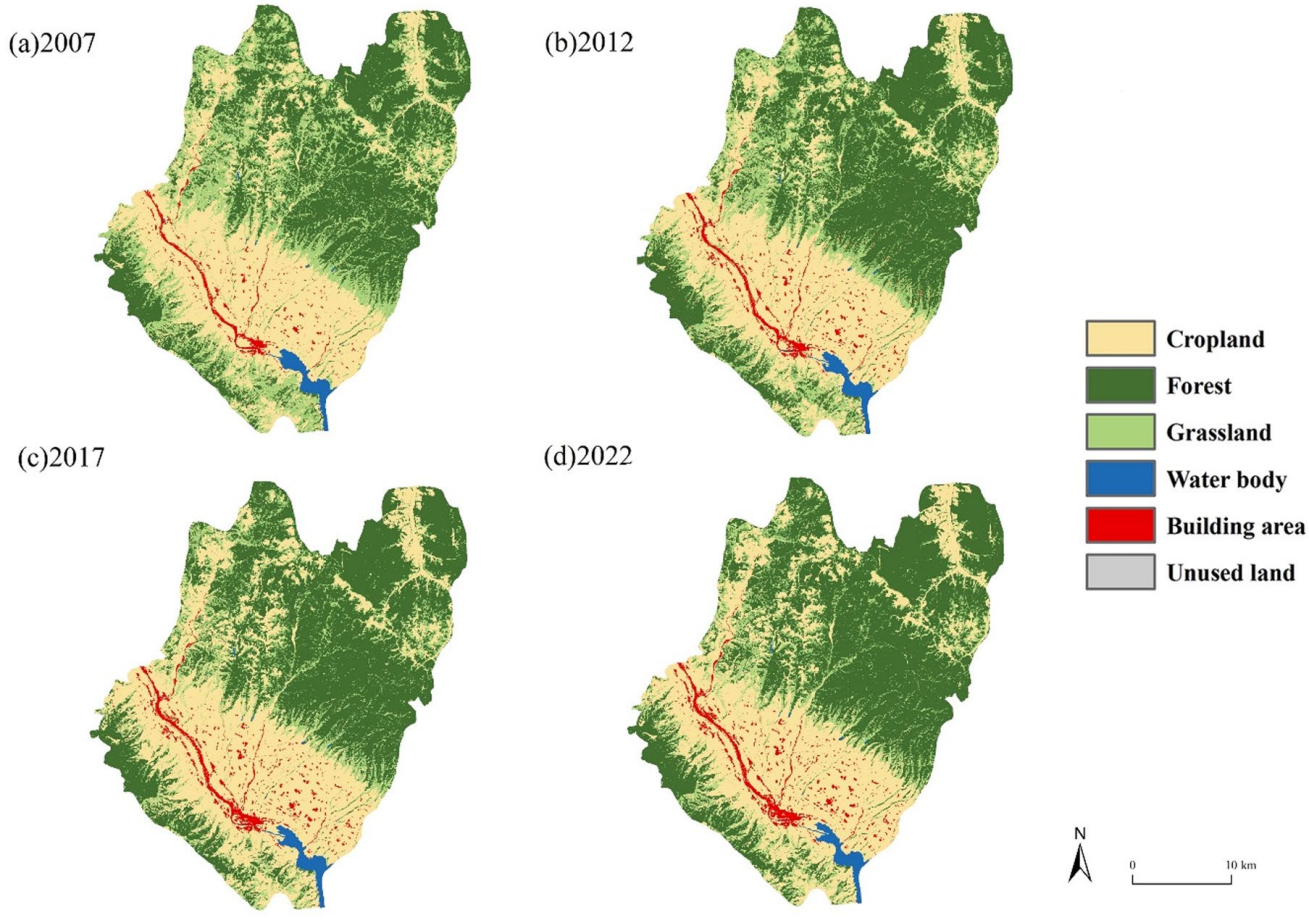
Land cover classification for 2007 (**a**), 2012 (**b**), 2017 (**c**) and 2022 (**d**). The administrative boundaries were obtained from the National Geographic Information Public Service Platform (https://map.tianditu.gov.cn). The maps were generated using ArcGIS Pro software version 3.2 (ESRI: https://www.esri.com/en-us/arcgis/products/arcgis-pro/overview).

The LULC maps from 2007 to 2022 showed that the main land cover type in Qianyang County is forest, accounting for more than 45% of the total study area (Fig. 3). During the last 15 years, forest was the land type that had the most changes in Qianyang County, with a total increase of 80.7 km^2^. Using the zonal statistics of satellite images in 2007, 2012, 2017 and 2022, the land use area data of Qianyang County at four dates are presented in Table 4.

**Table 4:** Land use area of Qianyang County (km^2^).

Land type	2007		2012		2017		2022	
	Area	%	Area	%	Area	%	Area	%
Cropland	350.98	35.22	331.80	33.30	374.38	37.57	390.78	39.22
Forest	371.47	37.28	414.92	41.64	434.03	43.56	452.17	45.38
Grassland	243.20	24.41	215.35	21.61	150.35	15.09	114.97	11.54
Water body	12.52	1.26	12.35	1.24	12.17	1.22	12.06	1.21
Building area	16.15	1.62	20.67	2.07	24.55	2.46	26.09	2.62
Unused land	2.14	0.21	1.37	0.14	0.98	0.10	0.40	0.04
Total	996.46	100.00	996.46	100.00	996.46	100	996.46	100

**Fig. 4:**
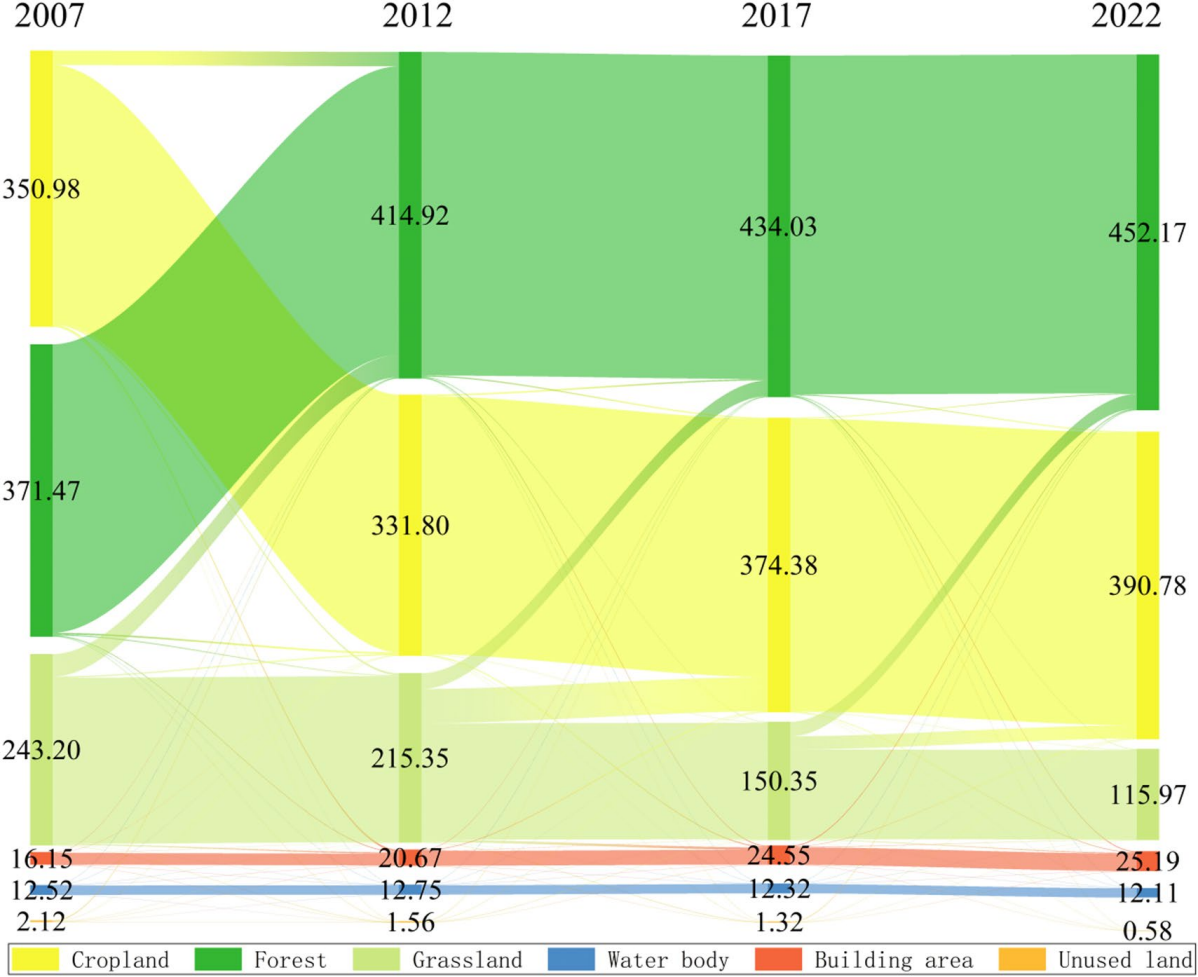
Sankey chart representing land use land cover changes. The map was generated using OriginPro 2022 (OriginLab https://www.originlab.com/).

The changes in grassland, water, and unused land in Qianyang County over the past 15 years were negative overall, with decreases of − 12.87%, − 0.05%, and − 0.17%, respectively. The areas represented by these three land types have been decreasing, with the change rate of grassland area being the largest (Table 5 & Fig. 4). In contrast, the changes in cropland, forest, and building area were positive, at 4%, 8.1%, and 1%, respectively. This indicates that cropland land, forest, and building area exhibited an increasing trend during the study period. From 2007 to 2022, the cropland experienced both positive and negative changes, but the overall reduction in cropland was greater than the increase.

**Table 5:** Land use change in three periods of Qianyang County (%).

Land type	2007–2012	2012–2017	2017–2022	Changes
Cropland	− 1.92	4.27	1.65	1.71
Forest	4.36	1.92	1.82	8.68
Grassland	− 2.79	− 6.52	− 3.55	− 11.15
Water body	− 0.02	− 0.02	− 0.01	− 0.05
Building area	0.45	0.39	0.15	1.06
Unused land	− 0.08	− 0.04	− 0.06	− 0.25

### Analysis on ESV changes

According to Table 6, the per unit area ESV is the highest in water bodies, followed by forest land, grassland, arable land and unused land. The ESV situation in Qingyang County from 2007 to 2022 is shown in Table 7. Over the past 15 years, the ESV has exhibited an initial growth followed by a decline, ultimately increasing from 1531.51 × 10^6^ yuan in 2007 to 1539.94 × 10^6^ yuan in 2022, with a net increase of 8.43 × 10^6^ yuan.

**Table 6:** ESV per unit area in Qianyang County (Yuan/km^2^).

Ecosystem services		Cropland	Forest	Grassland	Water body	Building areas	Unused land
Supply services	1	1053.85	289.29	289.29	812.09	0.00	12.40
	2	495.93	665.38	425.67	452.54	0.00	37.19
	3	24.80	343.02	235.57	6744.67	0.00	24.80
Regulation services	4	830.69	2182.10	1496.06	1655.17	0.00	136.38
	5	446.34	6529.77	3955.06	3651.30	0.00	123.98
	6	123.98	1942.40	1305.95	5672.22	0.00	384.35
Support services	7	334.75	4723.75	2897.07	78400.60	0.00	260.36
	8	1277.02	2657.37	1822.55	2008.52	0.00	161.18
	9	148.78	202.51	140.51	154.98	0.00	12.40
	10	161.18	2421.80	1657.24	6459.51	0.00	148.78
Cultural services	11	74.39	1062.12	731.50	4103.83	0.00	61.99
Total		4971.72	23019.5	14956.47	110115.4	0.00	1363.81

NB: ES secondary categories = 1: Food production; 2: Raw material production; 3: Water supply; 4: Gas regulation; 5: Climate regulation; 6: Waste disposal; 7: Hydrological regulation; 8: Soil conservation; 9: Maintain nutrient; 10: Biodiversity; 11: Aesthetic landscape.

**Table 7:** ESV valuation in Qianyang County from 2007 to 2022.

Land type	2007		2012		2017		2022	
	10^6^ yuan	%	10^6^ yuan	%	10^6^ yuan	%	10^6^ yuan	%
Cropland	174.50	11.39	164.96	10.45	186.13	12.05	194.29	12.62
Forest	855.12	55.83	955.13	60.51	999.11	64.70	1040.87	67.59
Grassland	363.74	23.75	322.09	20.41	224.87	14.56	171.95	11.17
Water body	137.87	9.00	136.00	8.62	134.00	8.68	132.78	8.62
Building area	0.00	0.00	0.00	0.00	0.00	0.00	0.00	0.00
Unused land	0.29	0.02	0.19	0.01	0.13	0.01	0.05	0.00
Total	1531.51	100	1578.36	100	1544.25	100	1539.94	100

1 km^2^ = 100 ha; 1 CNY ≈ 0.14 USD (United States Dollar).

The changes in ESV are influenced by the regional land use structure (Table 7). From 2007 to 2022, the area of cropland and forest land has consistently increased, while the area of grassland and unused land has decreased, and the area of water body has remained stable. Despite increases in cropland (39.78 km^2^) and forest (80.7 km^2^) areas, the overall ESV rise was constrained by grassland loss (− 128.23 km^2^). This occurs because grassland has higher per-unit ESV (14,956.47 yuan/km^2^) compared to cropland (4,971.72 yuan/km^2^). Therefore, the conversion of high-value grassland to lower-value land types resulted in net ESV loss that partially offset gains from forest expansion.

### Distribution of ESV cold/hot spots

**Fig. 5:**
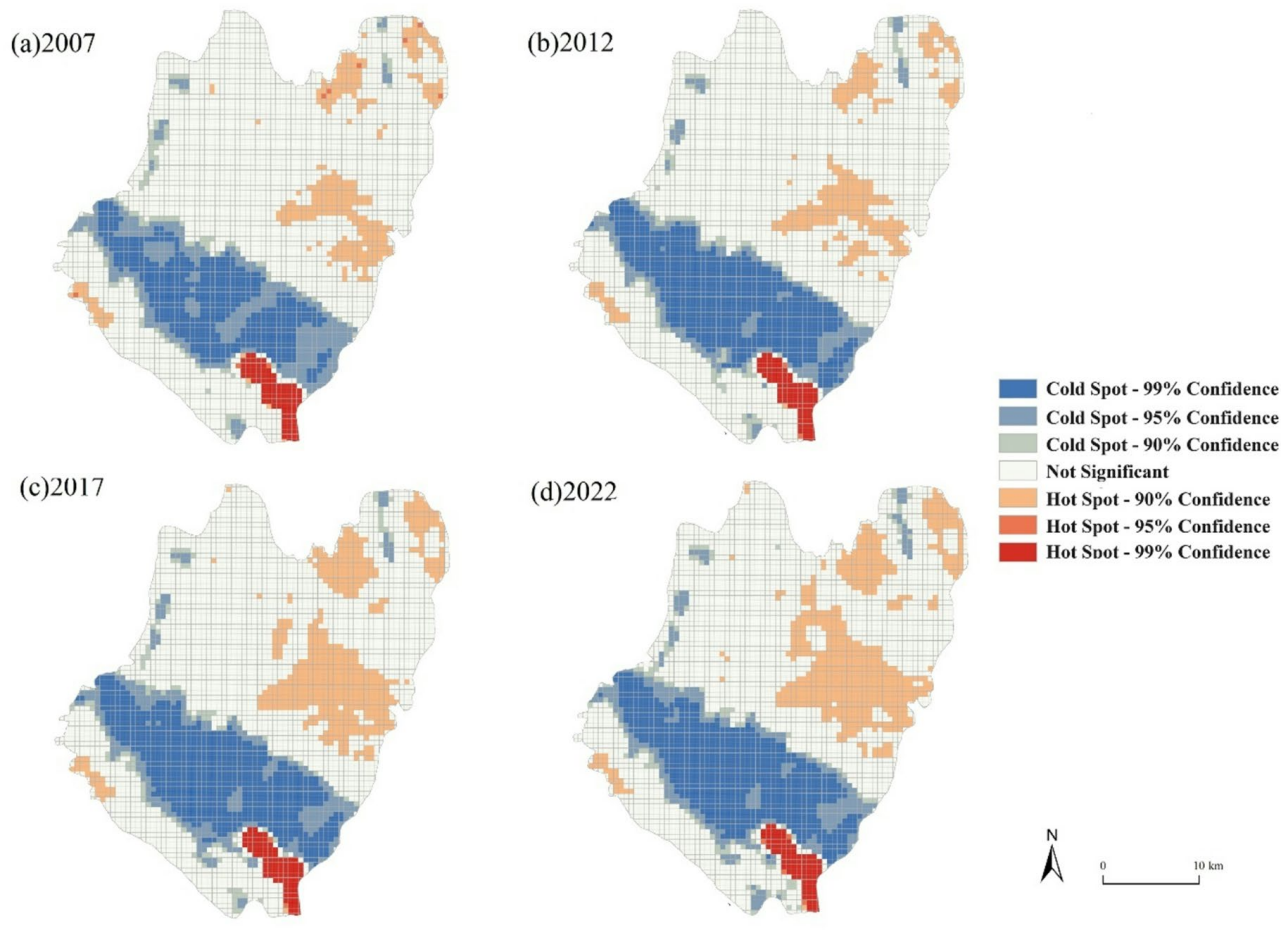
ESV Cold and Hot Spot Maps of 2007 (**a**), 2012 (**b**), 2017 (**c**) and 2022 (**d**). The administrative boundaries were obtained from the National Geographic Information Public Service Platform (https://map.tianditu.gov.cn). The maps were generated using ArcGIS Pro software version 3.2 (ESRI: https://www.esri.com/en-us/arcgis/products/arcgis-pro/overview).

Hot and cold spots indicated the change of the ESV. The warmest colour and coldest colour represented high and low ESV per unit as shown in the Fig. 5.

The cold spot area is primarily concentrated in the southern part of the central region, while the hot spot area is scattered. The largest area consists of non-significant points, primarily located in the northern and south-western regions of the central area. Between 2007 and 2012, the hot spot area experienced a gradual increase, while the total coverage of cold spots remained stable. Notably, some cold spots transformed into extremely cold spots during this period. From 2012 to 2017, the north-eastern area of the county witnessed steady growth in the hot spot area, while the original non-significant points gradually shrank and transformed into hot spots. From 2017 to 2022, there was a continuous expansion of the hot spot area, with minimal changes observed in the cold spot area. Meanwhile, the area occupied by non-significant points gradually decreased.

### LULC and ESV changes in multiple scenarios

The simulation of land change scenarios used the following conditions:

1. Natural development scenario. This type of scenario is based on the LULC law of the research area from 2015 to 2020 and is set without intervention in the conversion rules according to the existing urbanization development model and land use conversion rate.
2. Ecological protection scenarios. This scenario prioritizes ecological benefits as the urban development goal, strengthens ecological protection efforts, and reduces disorderly urban expansion. The probability of conversion from forest, grassland, and water body to building area is reduced by 80%, the probability of conversion from cropland and building area to forest increased by 50%, and the probability of conversion to grassland and water body increased by 35%.
3. Development priority scenario. Considering the further promotion of policies related to urban development and county-level construction in the research area, in the urban development scenario, the probability of cropland transferring to building area increases by 80%, the probability of forest and grassland transferring to building area increases by 60%, and the probability of construction lands transferring to other land decreases by 50%.

**Fig. 6:**
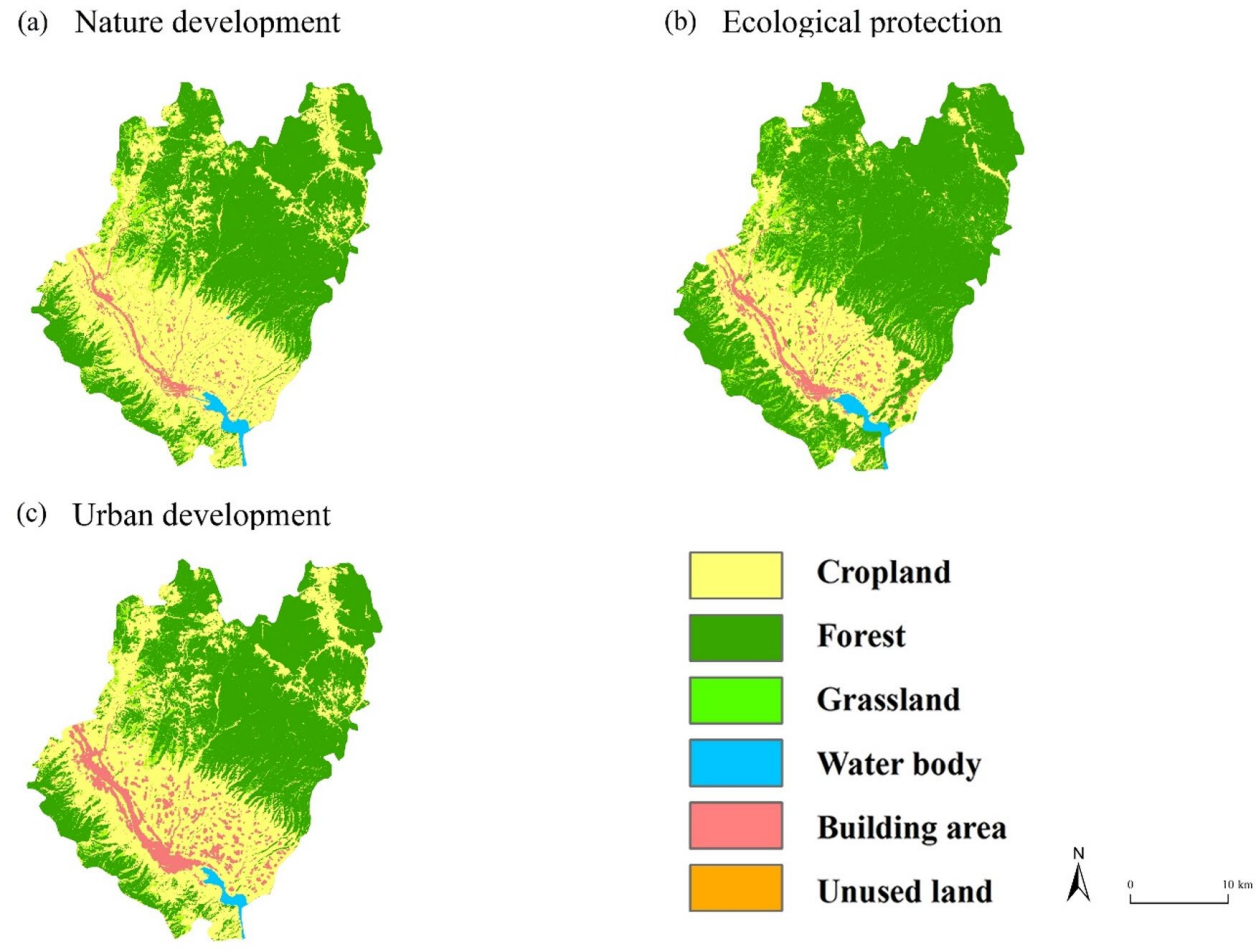
ESV under the 3 Scenarios. The maps were used the PLUS model (High Performance Spatial Computational Intelligence Lab, China: https://www.urbancomp.net/archives/plus) for simulation and generated using ArcGIS Pro software version 3.2 (ESRI: https://www.esri.com/en-us/arcgis/products/arcgis-pro/overview).

In 2050, the ESV of the study area under the scenarios of natural development, ecological protection, and urban development are shown in Fig. 6 and Table 8.

**Table 8:** Different land use types in 2050 under different scenarios.

Land type	2012		Natural development by 2050		Ecological protection by 2050		Urban development by 2050	
	Area	%	Area	%	Area	%	Area	%
Cropland	164.96	10.45	195.20	12.5367	122.73	6.8145	191.39	12.9265
Forest	955.13	60.51	1161.66	74.6081	1476.56	81.9831	1143.71	77.2480
Grassland	322.09	20.41	93.52	6.0061	91.77	5.0953	64.91	4.3842
Water body	136.00	8.62	106.63	6.8481	109.99	6.1069	80.55	5.4407
Building area	0.00	0.00	0.00	0.0000	0.00	0.0000	0.00	0.0000
Unused land	0.19	0.01	0.01	0.0009	0.00	0.0002	0.01	0.0006
Total	1578.36	100	1557.02	100	1801.06	100	1480.57	100

Compared to the ESV in Qianyang County from 2007 to 2022, the overall ESV is higher in 2050 under the natural development scenario. This is the highest ESV among the years 2007–2022. In 2050, the overall ESV is much higher under the ecological protection scenario than under the natural development and urban development scenarios. The urban development scenario has the lowest ESV. The overall ESV decreased from 1540*10^6^ in 2017 (see Table 7) to 1480.57*10^6^ in 2050 (Appendix A). In 2050, the cropland areas are 392, 246, and 384 km^2^, respectively, under the three scenarios (Appendix B). The ESV of forests increases significantly, making it the largest among the six land-use types (Table 8). The forest land has the largest area under the ecological protection scenario. Compared to the natural development and urban development scenarios, the forest ESV under the ecological protection scenario is higher by 31.49 × 10^6^ yuan and 33.28 × 10^6^ yuan, respectively. The ESV of water body under the ecological protection scenario is higher by 0.336 × 10^6^ yuan and 2.94 × 10^6^ yuan, respectively. The ESV difference for unused land is small among the three scenarios. These results show that the percentage of grassland declined from 20.41% in 2012 to 4.384% in 2050, while the percentage of water decreased from 8.62% to 5.44%. In contrast, the percentage of forests increased from 60.51% to 77.25%.

### Driving factor analysis

The LEAS module plays a crucial role in the PLUS model by identifying and analyzing the driving factors of land use change. This module integrates land use data with relevant socio-economic and natural environmental data to uncover the key factors influencing land use change. Due to the small study area, we did not choose NDVI (Normalized Difference Vegetation Index). The data used is from 2010 to 2020, as we are examining the impact of factors on land use based on past land use changes and linking these factors to ESV changes.

**Fig. 7:**
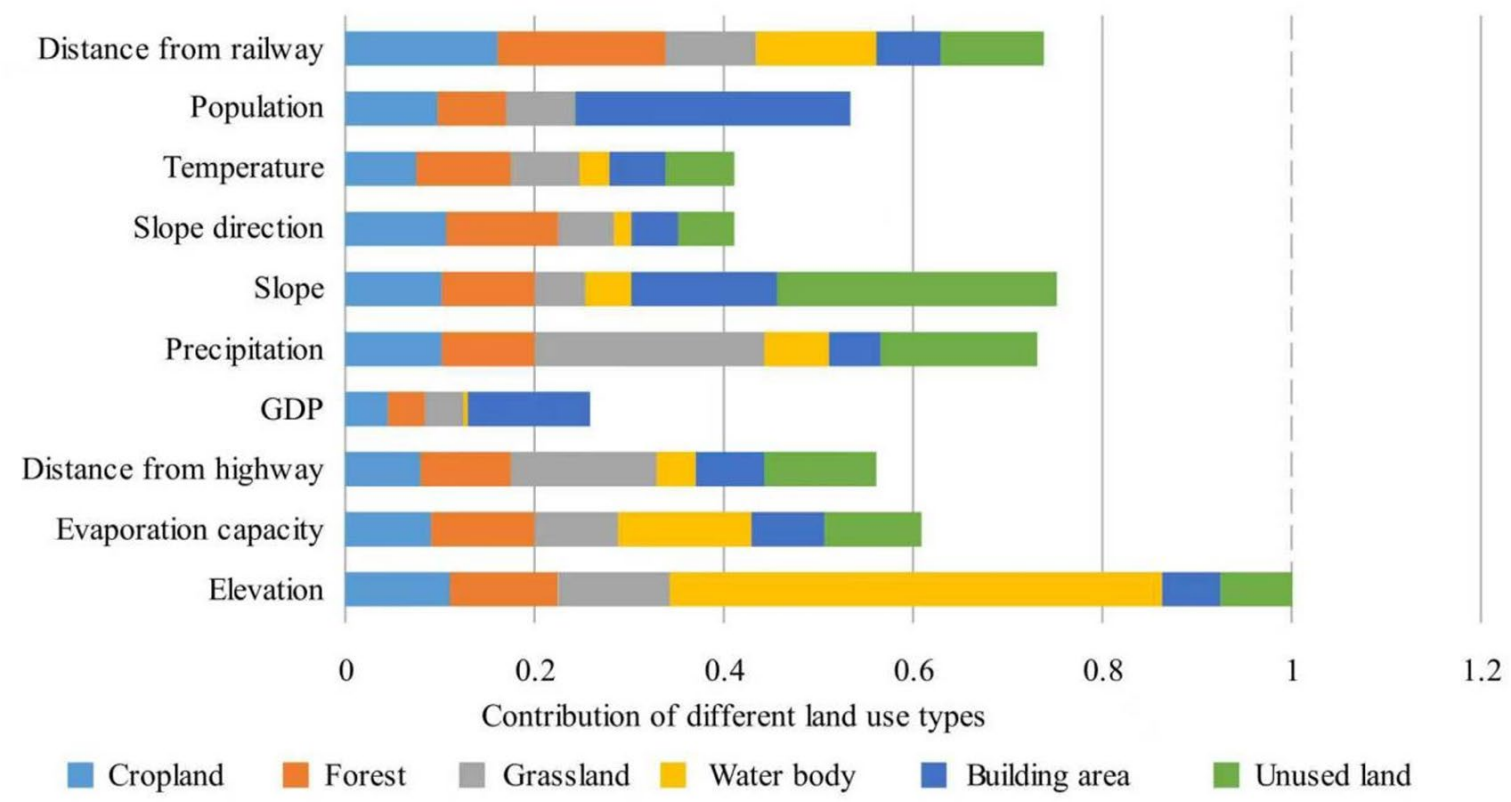
ESV Contribution of different land types factors. X-axis: contribution of driving factors to LULC change (%) for each land type. Created using the software Microsoft Excel 365.

From the perspective of cropland and grassland, the distance to railways has the greatest impact. Railways, as a leading infrastructure for urbanization, can cause significant changes in cropland as urbanization extends to suburban areas. Slope affects whether land is used for farming or forests. Slope direction affects the movement and distribution of water, hydrological cycle, water retention capacity of land, mechanical cultivation conditions of arable land, and development of forests. Elevation also contributes significantly, indicating that terrain characteristics have a significant impact on the distribution of grassland. In terms of water body, the distribution and changes of rivers and reservoirs in Qianyang County are closely related to terrain conditions. Precipitation and evaporation may also affect the distribution and size of water body, as the amount of precipitation directly determines the water volume of rivers and lakes. Adequate precipitation is a key factor affecting grassland. The building and unused land areas are small (Fig. 7). Looking at population, slope, and GDP, population growth leads to increased housing demand and drives the urbanization process. The driving factors identified the main changes that lead to ESV increasing and decreasing. Random forest analysis quantified each factor’s importance through variable importance scores. The model achieved R^2^ > 0.85 for all land types, indicating strong explanatory power.

### Validation of the PLUS model simulation

**Fig. 8:**
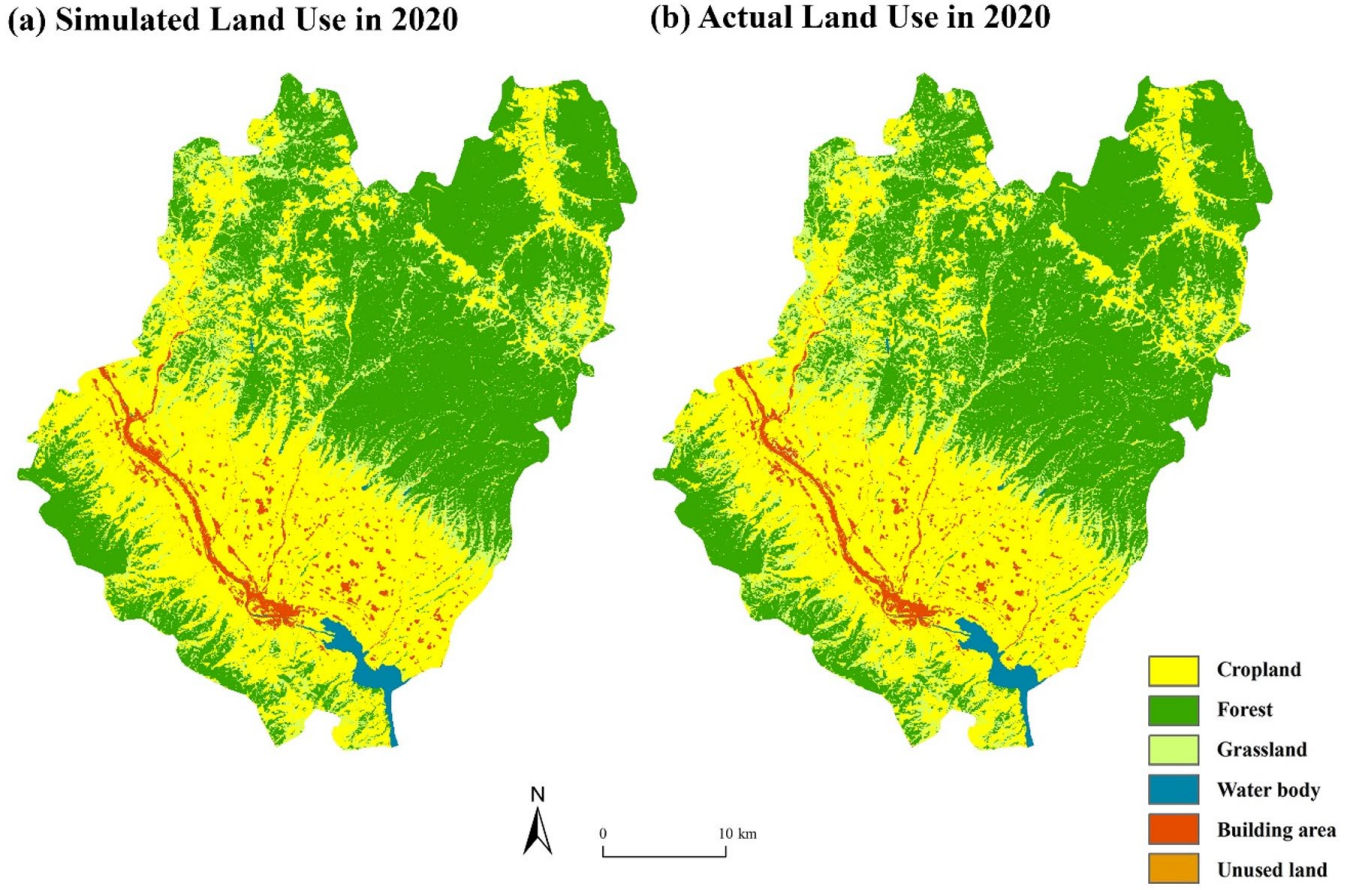
Comparison of Simulated (**a**) and Actual Land (**b**) Use Types in 2020. The maps were created using the PLUS model (High Performance Spatial Computational Intelligence Lab, China: https://www.urbancomp.net/archives/plus) for prediction and generated with ArcGIS Pro software version 3.2 (ESRI: https://www.esri.com/en-us/arcgis/products/arcgis-pro/overview).

To ensure the reliability and accuracy of the model in predicting future land use, a comparison was made between the actual land cover in the study area in 2020 and the simulated land cover (Fig. 8) to assess the model’s simulation accuracy. In this study, accuracy validation was performed by comparing the simulated results for 10 and 15 years with the actual results for 20 years. Due to the limited amount of unused land in 2017, it was not possible to sample this land use class, so it was replaced to reflect the simulation accuracy.

We compared the simulated and actual results for accuracy assessment. Model validation adhered to international best practices for land use change modeling^52,53^, employing temporal cross-validation where 2015 data were used to predict 2020 patterns. Although not directly using global frameworks (Intergovernmental Panel on Climate Change (IPCC), World Bank), the PLUS model integrates land use and driving factor data through the LEAS module and simulates spatial distribution through the CARS module, which can effectively capture land use change trends and provide support for ecosystem service value analysis.

Further analysis of the samples reveals different characteristics in the Producer’s Accuracy and User’s Accuracy for various land use types in Qianyang County (see Table 9). Both arable land and forest exhibit high levels of Producer’s Accuracy and User’s Accuracy. The Producer’s Accuracy for arable land is 93.02%, and the User’s Accuracy is 90.59%, both exceeding the threshold of 90%, indicating a high proportion of correct identification of arable land during the classification process. The Producer’s Accuracy and User’s Accuracy for forest are even higher, reaching 95.30% and 95.46% respectively. This is mainly attributed to the strict management and protection of arable land and forest in Qianyang County, making their characteristics clearly distinguishable in remote sensing images.

**Table 9:** Producer’s accuracy and user’s accuracy.

Land use type	Cropland	Forest	Grassland	Water body	Building area	Unused land
Producer’s accuracy	93.02%	95.30%	61.42%	98.63%	90.89%	72.58%
User’s accuracy	90.59%	95.46%	68.15%	97.69%	87.41%	27.48%

On the other hand, the Producer’s Accuracy and User’s Accuracy for unused land are relatively low, at 72.58% and 27.48% respectively, both below the 80% level. This is primarily due to the small proportion and scattered distribution of unused land in Qianyang County, which makes it challenging to accurately identify in remote sensing images. Additionally, unused land may share similarities in image features with building area and water body, further complicating the classification process.

## Discussion

In this work, we evaluate ESV using the LULC changes noted in Qianyang County over a 15-year period. To provide a spatial perspective when assessing the distribution effects of ecosystem services in the landscape, we categorized and graded the ecological values of each land cover class. This study contributes with novel information about ESV and scenarios for the study area.

The observed LULC changes demonstrate significant landscape transformation over the 15-year study period. Our findings align with global trends reported by Costanza et al.^41^, who observed ESV improvements in regions with forest expansion. The growth of forest area indicates improvements in the local ecological environment^54^, while the increase in building area and the significant decrease in cropland and unused land demonstrate that the urbanization process in Qianyang County has accelerated over the past 15 years. Most cropland, water body, and unused land are being converted into forest. This change is connected to the Ecological Welfare Woodland Program (EWFP) and the Cropland to Woodland Program (CCFP). In Qianyang County, these programs have directly improved the supply of resources (such as food, water, and biomass energy) as well as regulation and maintenance (such as controlling waste, flow, and the physical and biological environment). Additionally, Feng^55^ observed an increase in water yield by converting steeply sloping cropland to forest or grassland, as noted by Zhang^56^. It should be noted that the Chinese government has implemented a payment for ecosystem services (PES) plan.

Regarding ESV changes, our analysis reveals complex temporal dynamics. The ESV exhibited initial growth followed by a decline. Some researchers argue^57^ that one reason for the reduction in cropland is the rise of China’s middle class, as meeting the consumption standards of developed countries could double environmental impact globally. This increase directly affects housing demand and stimulates a construction boom. In countries like China, cropland is used not only for agriculture but also for housing due to better settlement conditions. The overall improvement in landscape and biodiversity indicators can be attributed to the expansion of forest during the study period, particularly from 2017 to 2022. Qianyang County has adjusted its land use structure and achieved remarkable results in ecological civilization construction, which mirrors international reforestation efforts^19^.

The spatial distribution patterns of ESV hotspots and coldspots provide insights into ecosystem service clustering. Additionally, the main reasons for the loss of ESV are related to environmental damage and the increase in building area, along with high population density; however, such areas have been shrinking. The area of ESV value-added hotspots in Qianyang County has been expanding, further illustrating the development of the ecological environment in the region. Additional analysis is required to understand the causes of spatial aggregation and the cold and hot spots presented in this article.

The multiple scenario analysis for 2050 reveals important policy implications. In 2050, the ESV of the study area under the scenarios of natural development, ecological protection, and urban development is presented in Appendix B. Compared to 2012, there is a decrease of 21.35 × 10^6^ yuan under the natural development scenario, an increase of 22.270 × 10^6^ yuan under the ecological protection scenario, and a decrease of 97.79 × 10^6^ yuan under the urban development scenario. These changes can be attributed to the decrease in forests and water body, and the increase in cropland. Overall, although there have been significant differences in the spatial pattern of ESV in Qianyang County over the past 15 years, regional agglomeration, uniformity, and connectivity have strengthened, while the degree of fragmentation and landscape dominance has decreased.

From the perspective of driving factors, our analysis identifies key variables influencing land use change. Urbanization-driven cropland conversion, as observed here, is a classic example of direct human pressure on ES, as categorized by Fisher^58^. It is inevitable that cropland, grassland, and water body will be converted into building area in a rapidly expanding economy with a dense population and a burgeoning middle class. Xie et al.^42^ noted that in economically developed and densely populated areas, the ratio of ESV per capita gross domestic product (GDP) decreases. This informs Qianyang County that, in the future, when converting agricultural land to urban areas, it must minimize negative environmental effects.

Concerning model validation and limitations, several considerations warrant attention. The image classification process required manual intervention in 3 key steps: (1) removing small patches via majority/minority filtering, (2) correcting unreasonable classifications (e.g., reclassifying non-water pixels in rivers) using rule sets, and (3) manually editing misclassified areas. These interventions were applied uniformly across all four time-period datasets (2007, 2012, 2017, 2022) to ensure consistency. While manual correction enhanced accuracy, it introduced some subjectivity which we tried to mitigate via consistent rules. The PLUS model is influenced by both subjective and objective factors in this study. The selection of influencing variables primarily focuses on natural, environmental, and socio-economic factors, which are closely related to the simulation results. However, the choice of driving factors may be affected by the subjectivity of researchers and limited data availability. Additionally, urban land use changes are heavily influenced by policy planning orientations, which may increase the uncertainty of simulation results. Therefore, future research should ensure the reasonable updating of region-specific driving factors. Another limitation of the ESV assessment is the reliance on national-level coefficients^42^, which may not fully capture regional heterogeneity in ecosystem quality. Additionally, the exclusion of cultural ESV for construction land (due to low sensitivity coefficients) may underestimate the comprehensive ESV of urbanized regions, as cultural services like recreational value are not quantified.

There is room for improvement in the accuracy and timeliness of the ESV estimation model. The calculation method of ESV equivalents proposed by Xie^42^ is based on national circumstances. Considering the differences in structure and quality of the same ecosystem type across different climatic regions in China can lead to uncertainty in the ESV coefficients, with different land use types corresponding to different magnitudes of ESV. Xie’s value estimates for woodlands and croplands are much larger than those calculated by Costanza^9^ for wetlands and water bodies, indicating a high degree of heterogeneity in ESV. A single set of ESV coefficients cannot adequately measure the value of ecosystem services in every region. However, such studies have not considered the heterogeneity of ecosystem services caused by uneven land use distribution in specific areas, nor the internal relationship between ESV and land use change, which could not precisely characterize the subtle differences in the ecological services provided by the dynamic changes of specific land use types.

Biomass, vegetation coverage, and net primary productivity can be used to revise the coefficients to modify ESV, effectively reflecting the land use types of forest and cropland with substantial vegetation coverage. However, this revision method is not applicable to unused land (e.g., bare land with sparse vegetation) and diverse wetlands (e.g., marshlands vs. riverine wetlands), even though these ecosystems also provide ES such as soil retention (unused land) and water purification (wetlands). Furthermore, additional analysis is required to understand the causes of spatial aggregation and the cold and hot spots presented in this article.

## Conclusions

This research indicates that forest, cropland, and grassland are the primary types of land use contributing to ESV, as determined by spatial autocorrelation analysis conducted in the studied area. We discovered a clustered distribution of ESV, where high-value areas in the centre spread outward from high to low. Urban population distribution, topographic conditions, and precipitation closely influence the ESV. Studying just one type of ES does not show the full picture of changes in the environment; so, we need to look at how things are spread out and change over time based on related studies to guide smart land use and ecological development in Shaanxi. This type of analysis can help us build a model for optimizing land use that simultaneously considers ecological conservation, especially of water bodies, while restraining urban expansion and minimizing non-intensive land use for ecological reasons. Additionally, by studying the impact of land use change on ESV under different scenarios, this paper emphasizes that, for land use management, the government must balance the relationship between urban development, agricultural land use, and environmental protection.

## Supplementary Information

Below is the link to the electronic supplementary material.

Supplementary Material 1

Supplementary Material 2

## Data Availability

The datasets used and analysed during the current study are available from the corresponding author on reasonable request.
