# Supplementary figures and images for: Land use and land cover changes drive ecosystem services value in the Chinese county of Qianyang
Source: Sci Rep. 2025 Dec 12;16:253. doi: 10.1038/s41598-025-29320-8 (PMC12770534; doi:10.1038/s41598-025-29320-8)

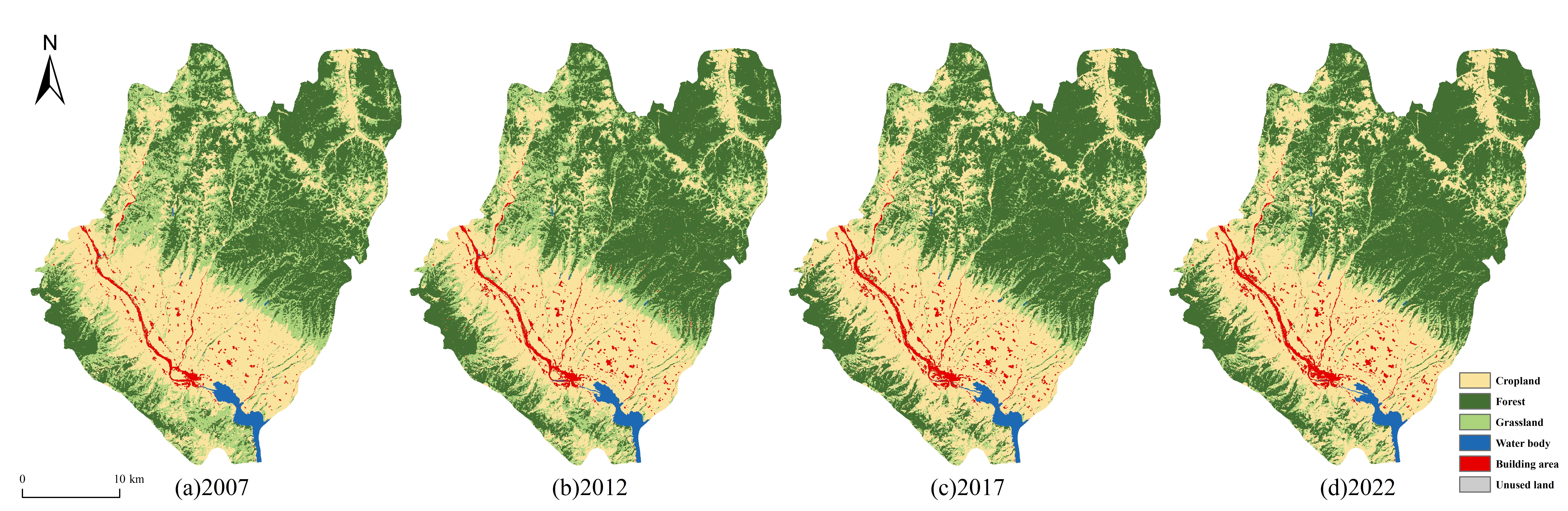

Supplement: Supplementary file 1 — Supplementary Material 1 [file 41598_2025_29320_MOESM1_ESM.zip › original Data 20107-2022/新建文件夹/lucc2007-2022.jpg]

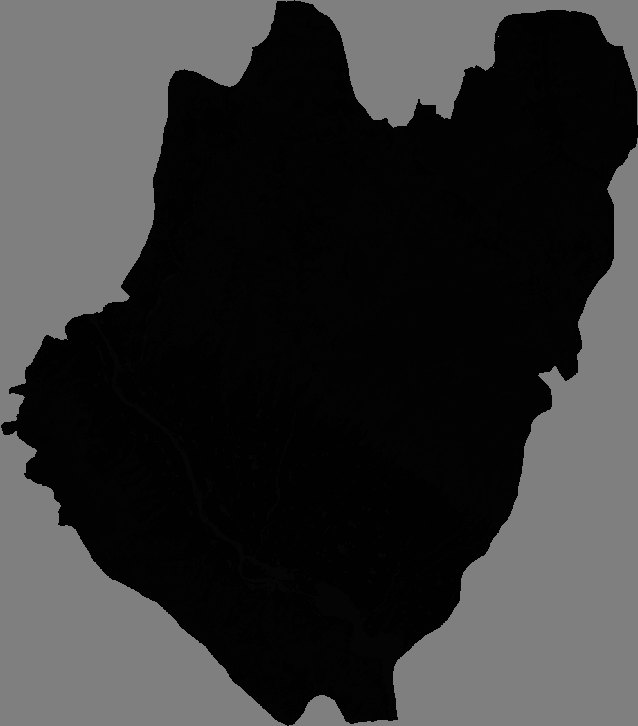

Supplement: Supplementary file 1 — Supplementary Material 1 [file 41598_2025_29320_MOESM1_ESM.zip › original Data 20107-2022/新建文件夹/lucc2007.tif.ovr]

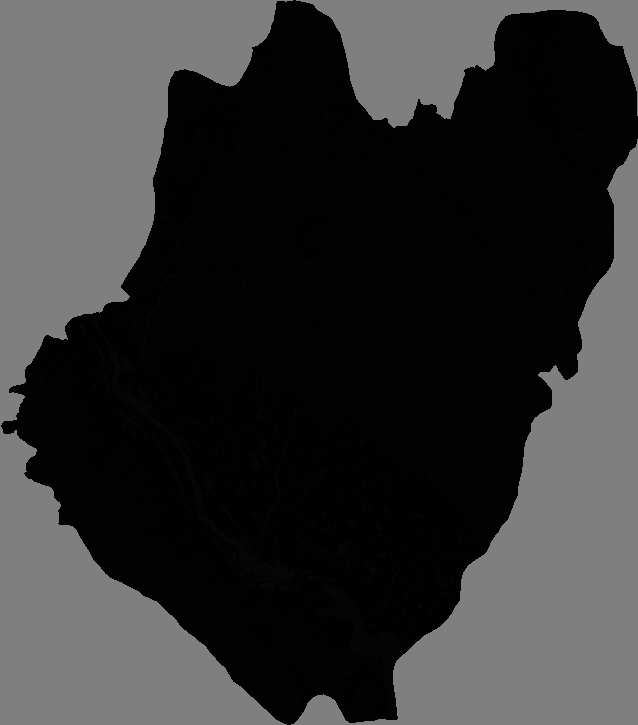

Supplement: Supplementary file 1 — Supplementary Material 1 [file 41598_2025_29320_MOESM1_ESM.zip › original Data 20107-2022/新建文件夹/lucc2022.tif.ovr]
