# Supplementary material for: Land use and land cover changes drive ecosystem services value in the Chinese county of Qianyang
Source: Sci Rep. 2025 Dec 12;16:253. doi: 10.1038/s41598-025-29320-8 (PMC12770534; doi:10.1038/s41598-025-29320-8)
Supplement: Supplementary file 2 — Supplementary Material 2 [file 41598_2025_29320_MOESM2_ESM.docx]

**Appendix A**

| Land use (in km2) | Cropland | Forest | Grassland | Water body | Building area | Unused land |
| --- | --- | --- | --- | --- | --- | --- |
| Natural development | 392.62 | 504.64 | 62.53 | 9.68 | 26.89 | 0.11 |
| Ecological protection | 246.86 | 641.44 | 61.36 | 9.99 | 36.78 | 0.03 |
| Urban development | 384.95 | 496.84 | 43.40 | 7.32 | 63.88 | 0.07 |

**Appendix B**

| Land use (in km^2^) | Cropland | Forest | Grassland | Water body | Building area | Unused land | Total |
| --- | --- | --- | --- | --- | --- | --- | --- |
| Natural development | 1951986.98 | 11616595.23 | 935160.17 | 1066266.72 | 0.00 | 143.69 | 1557015279.05 |
| Ecological protection | 1227327.65 | 14765644.06 | 917699.04 | 1099896.13 | 0.00 | 34.96 | 1801060183.57 |
| Urban development | 1913867.47 | 11437118.22 | 649105.44 | 805537.73 | 0.00 | 92.65 | 1480572150.01 |
